# A Rare Case of Septic Arthritis Secondary to Actinomyces europaeus Infection

**DOI:** 10.7759/cureus.112852

**Published:** 2026-07-17

**Authors:** Lauren Bathan, Sharjeel Ahmad

**Affiliations:** 1 Department of Medicine, University of Illinois College of Medicine Peoria, Peoria, USA

**Keywords:** actinomyces infection, acute septic arthritis, gram-positive bacteria, hand infection, native joint infection

## Abstract

*Actinomyces europaeus* is an uncommon cause of human infection and a rare cause of septic arthritis. We report an 81-year-old woman with diabetes mellitus, chronic kidney disease, gout, and dementia who developed septic arthritis of the distal interphalangeal joint of the right third finger during hospitalization for *Escherichia coli* bacteremia. Computed tomography demonstrated erosive joint destruction with an adjacent abscess. Surgical incision, drainage, and arthrotomy revealed septic arthritis with associated abscess formation and degeneration of the terminal extensor tendon. Operative cultures grew *A. europaeus* together with multiple anaerobic organisms. The patient underwent surgical debridement, followed by prolonged oral doxycycline therapy and a short course of metronidazole for polymicrobial anaerobic coverage, resulting in complete clinical resolution. This case highlights an uncommon cause of native joint septic arthritis and emphasizes the importance of considering unusual pathogens in patients with pre-existing joint disease and atypical clinical presentations.

## Introduction

Septic arthritis is a medical emergency associated with substantial morbidity and mortality if diagnosis and treatment are delayed. *Staphylococcus aureus* remains the most common causative organism, followed by streptococci and gram-negative bacilli, particularly among older adults and immunocompromised patients. Established risk factors include diabetes mellitus, chronic kidney disease, inflammatory arthritis, crystal arthropathies, immunosuppression, and pre-existing joint abnormalities [1,2].

Actinomyces species are facultative to obligately anaerobic, gram-positive bacilli that normally colonize the oropharynx, gastrointestinal tract, and female genital tract. Infection typically occurs following disruption of mucosal barriers and most commonly involves the cervicofacial, thoracic, abdominal, and pelvic regions. Although musculoskeletal involvement has been described, osteoarticular infections are uncommon, and septic arthritis caused by Actinomyces species is exceptionally rare [3,4].

*Actinomyces europaeus*, first described in 1997, is a relatively uncommon human pathogen distinguished from many other members of the genus by its lack of true filament formation and has most frequently been associated with skin and soft tissue infections [5,6]. To our knowledge, only one previous case of *A. europaeus* septic arthritis involving a prosthetic joint has been reported [7]. We describe what is, to our knowledge, the first reported case of native distal interphalangeal joint septic arthritis caused by *A. europaeus*.

## Case presentation

An 81-year-old female with a past med s mellitus, stage 4 chronic kidney disease, gout, and dementia initially presented to the hospital with altered mental status, visual hallucinations, decreased oral intake, generalized weakness, and foul-smelling urine. She was diagnosed with a urinary tract infection after her urinalysis demonstrated 3+ leukocyte esterase, positive nitrites, >100 white blood cells/high-power field, and many bacteria. She was found to have *Escherichia coli* bacteremia, with urine cultures showing growth of the same organism from samples obtained at admission. She was therefore placed on ceftriaxone 2 g every 24 hours intravenously for treatment. Repeat blood cultures obtained 48 hours after admission showed clearance of bacteremia, and no follow-up blood cultures were obtained during the remainder of the hospitalization. 

During her hospitalization, the patient began to experience right-hand pain, with no preceding mechanical injury or trauma. The physical examination showed significant swelling of the right hand and fingers, with the worst being the 3rd finger, with tenderness over its tip without erythema, drainage, an open wound, or necrosis. There was discoloration of the skin at the distal interphalangeal joint of the right middle finger, with some fluctuance. Sensation was grossly intact throughout the axillary, musculocutaneous, radial, median, and ulnar nerve distributions. Motor function was limited distally to fingers due to pain and swelling. Radial pulse was rated as 2+. Fingers were pink and warm, with capillary refill <2 seconds.

A right-hand X-ray was obtained, demonstrating soft-tissue swelling without acute osseous abnormality. She was afebrile. She had a normal white cell count on admission, but she subsequently developed leukocytosis. She had elevated fasting glucose, HbA1c, C-reactive protein, and erythrocyte sedimentation rate (Table 1). Antibiotic therapy was switched to oral levofloxacin 500 mg every 24 hours, and oral doxycycline 100 mg twice daily was added to the regimen two days later. A right-hand CT without contrast was obtained, demonstrating erosive changes within the distal interphalangeal joint of the right 3rd finger (Figure 1) with an adjacent 2.3 cm complex collection, suspicious for septic arthritis, an adjacent abscess, and cellulitis (Figure 2).

**Table 1:** Patient's pertinent lab values CRP, C-reactive protein; ESR, erythrocyte sedimentation rate; HbA1c, hemoglobin A1c; WBC, white blood cell.

	Patient’s lab level	Reference range
WBC	12.59 x 10^3^/µL	4.00-12.00 x 10^3^/μL
CRP	23.50 mg/dL	<0.50 mg/dL
ESR	>130 mm/h	<30 mm/h
Glucose (non-fasting)	112 mg/dL	<125 mg/dL
HbA1c	7.9%	4%-6%

**Figure 1:**
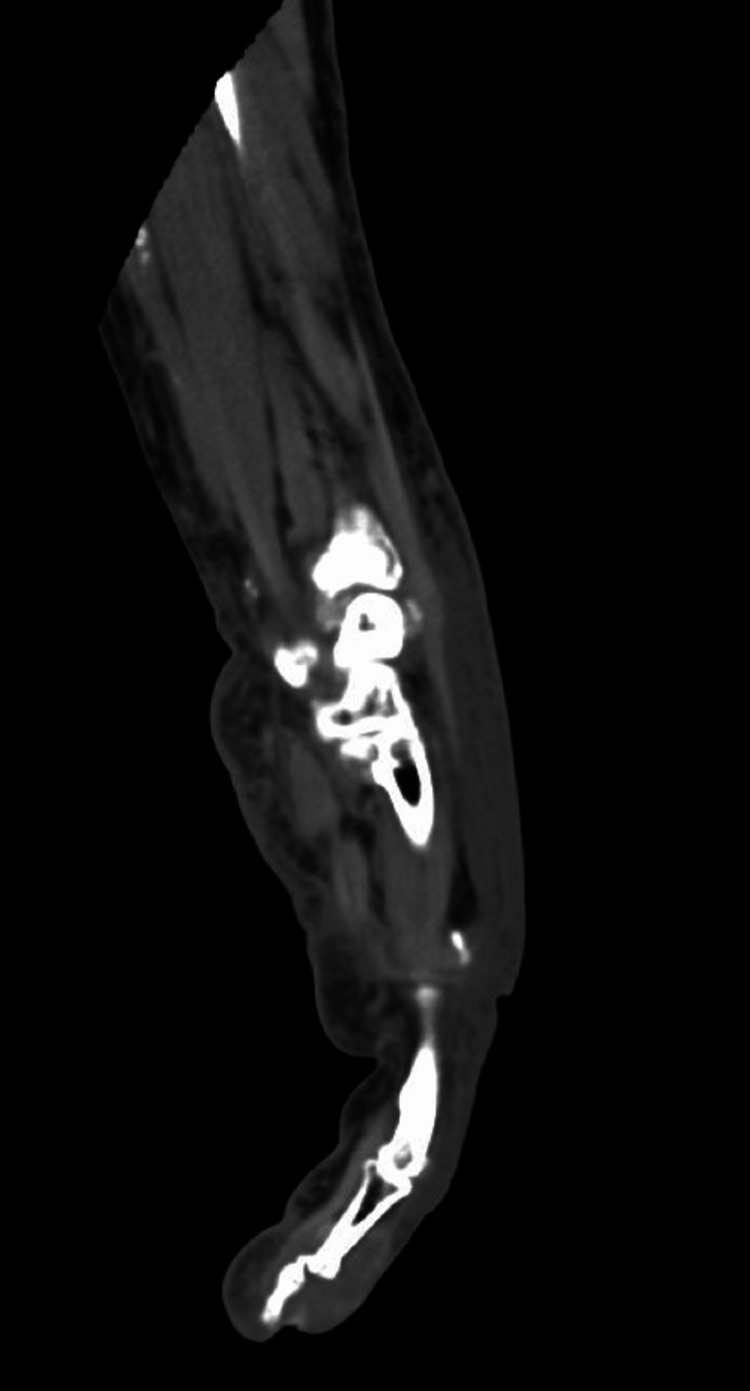
CT demonstrating erosive destruction of the right third distal interphalangeal joint. Sagittal CT image showing erosive destruction of the right third distal interphalangeal joint involving the distal and middle phalanges with adjacent soft tissue swelling, consistent with septic arthritis.

**Figure 2:**
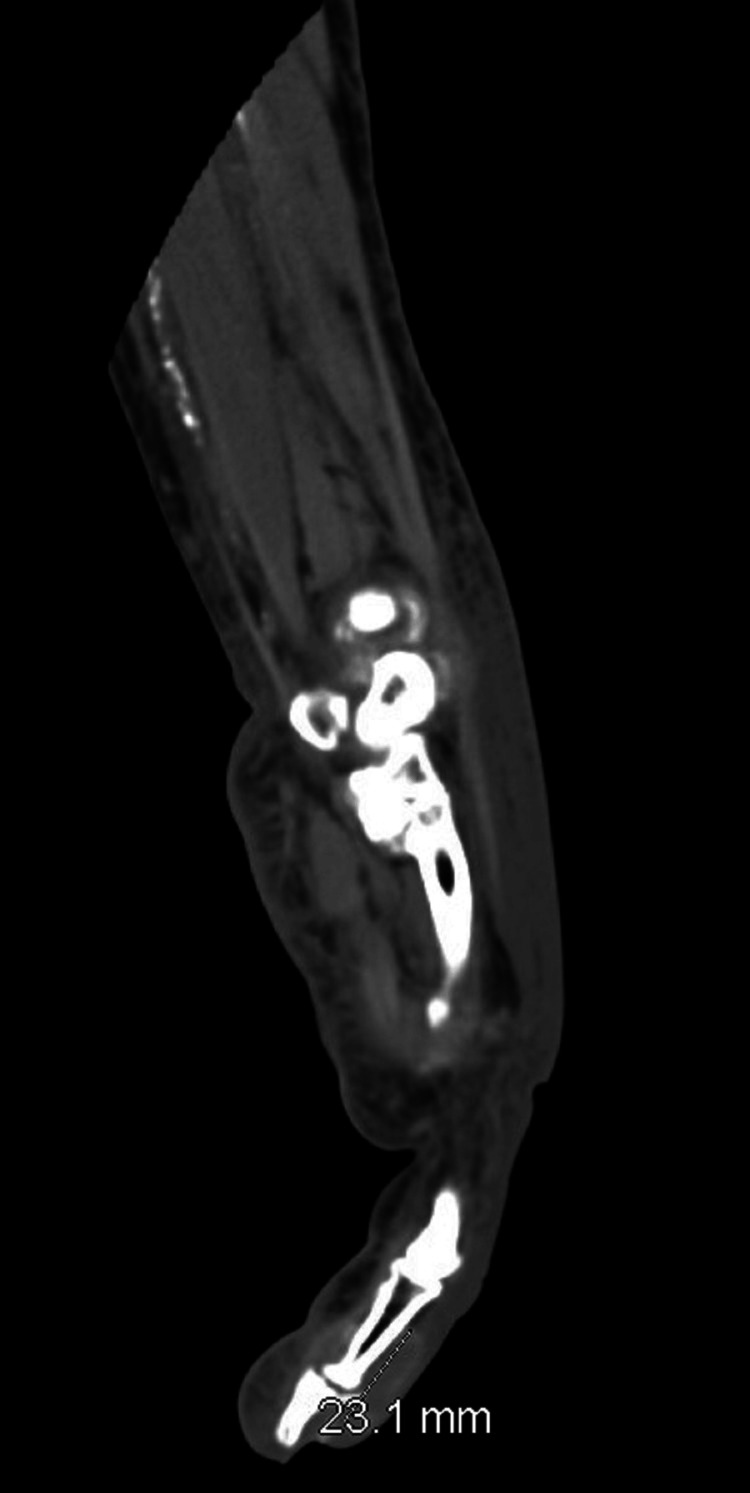
CT demonstrating periarticular abscess of the right third distal interphalangeal joint. Sagittal CT image showing a 23.1-mm complex periarticular fluid collection adjacent to the right third distal interphalangeal joint, compatible with an abscess.

The patient underwent incision and debridement of the right long finger, arthrotomy, and washout of the right 3rd distal interphalangeal joint, and cultures were obtained. Intraoperative findings included an abscess emanating from the distal interphalangeal joint down to the eponychial fold and evidence of septic arthritis and degeneration of the terminal extensor tendon. There was also a chronic nail deformity likely secondary to a mucous cyst, as well as evidence of gouty tophus/arthritis. From these findings, it was speculated that the mucous cyst likely became infected and then seeded into the already deformed and previously damaged (by gout) distal interphalangeal joint. Intraoperative cultures grew *A. europaeus* and multiple other anaerobic organisms (not further identified), and the Infectious Diseases service was consulted.

The patient was continued on oral doxycycline 100 mg twice daily for 6 months, levofloxacin was discontinued after a 8-day course, and a 14-day course of oral metronidazole 500 mg every 8 hours was added (to cover anaerobic organisms). Antibiotic susceptibility testing was ordered, with the specimen being sent to a reference lab, but unfortunately, a lab error occurred, and the wrong specimen was used, so those results were not available. She was discharged from the hospital on the above-mentioned regimen. She was seen in the Infectious Diseases clinic at three and six months. At completion of six months of doxycycline therapy, the patient demonstrated complete clinical resolution with normalization of inflammatory markers and no evidence of recurrent infection during follow-up.

## Discussion

Actinomyces species are facultative to obligately anaerobic, gram-positive, non-spore-forming, filamentous, non-motile bacteria that colonize the oropharynx, GI tract, and female genital tract, causing opportunistic infections with disruption of the mucosal barriers, as seen after trauma and surgical procedures, often as part of polymicrobial infection [3]. The infection can be quite indolent, developing slowly over time and often not presenting with the typical signs or symptoms. Infections are usually characterized by indurated “woody” lesions and the presence of “sulfur granules.”

*A. europaeus* was first described in 1997 as a newly proposed species within the Actinomyces genus [5]. The original study examined isolates from human abscess material, further analyzed by several European laboratories [6]. On solid media, the organism forms smooth, circular, translucent, grayish colonies, whereas under the microscope, it appears as short gram-positive rods with no true filament formation, a distinguishing feature of the species. Biochemically, the organism is also found to be non-motile, facultatively anaerobic, fermentative, and catalase-negative, consistent with the genus Actinomyces, though it does not hydrolyze esculin or ferment sucrose, which is also characteristic of this species. *A. europaeus* has often been co-isolated with coagulase-negative Staphylococcus spp. and Corynebacterium spp. and associated with skin and soft tissue infections involving the face, neck, chest, back, and genital areas, suggesting they may colonize the skin and subsequently cause infection [7]. It has also been detected in urinary tract infections, suggesting it may colonize the bladder and cause infection in this environment as well [3,5-6].

Our literature review (using PubMed and Google Scholar) revealed that *A. europaeus* joint infection has been reported only once previously. The case involved a 75-year-old female with a past medical history of obesity and rheumatoid arthritis who had undergone a left total knee arthroplasty within the year and presented with signs of inflammation, pain, and functional impairment. She was eventually diagnosed with an atypical periprosthetic joint infection post-total knee arthroplasty caused by *A. europaeus*. She was then treated with vancomycin and cefuroxime as well as two-stage reimplantation surgery [7].

In our case, the patient’s history of diabetes mellitus and joint destruction by gout predisposed her to the development of septic arthritis of her right 3rd distal interphalangeal joint from *A. europaeus*. Although intraoperative findings suggested infection originating from a chronic mucous cyst with extension into the damaged distal interphalangeal joint, alternative mechanisms should also be considered. Hematogenous seeding remains possible given the patient's concurrent *E. coli* bacteremia, although this appears less likely because *A. europaeus* rather than *E. coli* was recovered from operative cultures. Direct inoculation through minor, unnoticed trauma or colonization of chronically damaged tissue, followed by contiguous spread, is an additional plausible mechanism.

Penicillin G is the first-line treatment for Actinomycosis, typically administered as high-dose intravenous therapy for 2-6 weeks, followed by oral penicillin V or amoxicillin for a prolonged course of 2-12 months, depending on disease severity and location [8-10]. Alternative regimens for penicillin-allergic patients include tetracyclines (doxycycline), clindamycin, or erythromycin, all of which have demonstrated clinical efficacy [4]. Amoxicillin-clavulanate is also commonly used and may be preferred when beta-lactamase-producing co-pathogens are present, as actinomycosis is frequently polymicrobial [11]. They are almost uniformly susceptible to beta-lactams, carbapenems, and vancomycin but are resistant to metronidazole, which should be avoided despite its frequent empiric use in anaerobic infections. Fluoroquinolones, such as ciprofloxacin, also exhibit poor activity and should not be used [12]. Treatment duration should be individualized based on clinical response, disease location, and extent of infection. Susceptibility testing could not be completed because of a laboratory processing error, representing an important limitation of this report. However, published susceptibility data demonstrate that Actinomyces species generally remain susceptible to beta-lactams and tetracyclines.

Doxycycline was chosen for treatment in our patient for ease of antibiotic administration and lack of need for dose adjustment given her chronic kidney disease. Actinomycosis is frequently polymicrobial because disruption of mucosal or cutaneous barriers permits invasion by multiple anaerobic organisms. In the present case, *A. europaeus* was isolated alongside several anaerobic bacteria, making it impossible to determine the relative pathogenic contributions of each organism. Nevertheless, isolation of *A. europaeus* from operative specimens in conjunction with compatible intraoperative findings supports its role as a clinically significant pathogen. Although metronidazole lacks reliable activity against Actinomyces species, it was administered briefly to provide coverage for the accompanying anaerobic organisms identified intraoperatively until definitive treatment with doxycycline was continued.

Actinomyces joint infections are rare, reported in both native and prosthetic joints. Native septic arthritis most often involves the knee, sometimes following iatrogenic inoculation such as intra-articular injection, with *A. naeslundii* frequently implicated [13]. Prosthetic joint infections typically present as low-grade, indolent infections with delayed onset, sometimes years after implantation and often linked to hematogenous seeding from dental sources or IV drug use, involving species such as* A. israelii, A. neuii, *and* A. radingae* [14-17]. Diagnosis requires anaerobic transport and prolonged culture incubation, and molecular methods (16S ribosomal ribonucleic acid polymerase chain reaction (16S RNA PCR) and matrix-assisted laser desorption/ionization-time-of-flight mass spectrometry (MALDI-TOF MS)) may be necessary when cultures are negative [18,19].

Surgical intervention is often necessary in addition to antibiotics, particularly for abscess drainage, excision of fibrotic tissue, or removal of persistent sinus tracts. The combination of surgical debridement and antimicrobial therapy typically yields excellent outcomes, with cure rates exceeding 90% in most series [20]. 

## Conclusions

This case expands the limited literature describing osteoarticular infection caused by *A. europaeus* and illustrates that uncommon pathogens should remain in the differential diagnosis of septic arthritis, particularly in patients with diabetes mellitus and pre-existing joint abnormalities. Prompt surgical source control combined with prolonged targeted antimicrobial therapy can result in excellent clinical outcomes despite the rarity of this organism.
